# Digitized Industrial Work: Requirements, Opportunities, and Problems of Competence Development

**DOI:** 10.3389/fsoc.2020.00033

**Published:** 2020-06-09

**Authors:** Volker Baethge-Kinsky

**Affiliations:** Soziologisches Forschungsinstitut (SOFI), Göttingen, Germany

**Keywords:** digitalization, work, upskilling, job skills, competences, continuing education, learning at work, digital media

## Abstract

German companies have been affected by a new wave of digitalization during the past years, and this has led to responses both in the way production is organized and in the goals of the German industrial policy. The coordinated response is widely referred to as “Industry 4.0” and is intended to support German industries in the increasingly fierce competition for global leadership in manufacturing. Simultaneously, a social science debate about changes in work and in employee training began and continues to this day. Far-reaching predictions of fundamental change circulate, especially concerning the organization of work and worker competence requirements. Other issues include also the needs and opportunities of firm-based competence development at work as well as the role and the uses of digital media. In recent years, more empirical studies have become available for clarifying open questions, and this paper presents three main results from one such study based on case investigations in 10 German industrial firms. First, digitalization does not change industrial production work radically. There is no general trend of upskilling, downgrading, or reskilling. A moderate trend favoring upgrading is observable, however. Thus, traditional workforce competences are not becoming “obsolete.” Rather, in the context of automation, they are being complemented by other skills including new technical qualifications in information technology as well as the ability to take a more theoretical approach to problem-solving in process optimization. Second, our results confirm more cautious assessments of the need for accelerated continuing vocational training. They also make it clear that the potential for increased learning opportunities in digital work and for the increased use of digital media in continuing vocational training has been overestimated. Learning opportunities seem to decrease rather than increase with digital work. Moreover, the use of digital media in continuing vocational training is limited due to organizational, financial, and cultural constraints and due to the lack of knowledge about the effectiveness of digital learning environments. Third, a number of organizational measures are needed to manage change. One measure would be to integrate new skill requirements into a binding institutional curriculum for education and training. Another measure would be to make on-the-job learning opportunities a central aspect of how work is organized.

## Introduction

Like other industrialized countries within and outside Europe, Germany recently augmented its national industrial policy (Krzywdzinski, 2017, p. 245). “Industry 4.0” was intended to support companies in the intensified competition for global leadership in manufacturing. An essential component of this strategy is the establishment of new production concepts based on the networking of all processes via the internet, new digital assistance systems, and novel automation solutions. In this context, the promoters of Industry 4.0 emphasized the new role of the workforce as “conductors of technology” (Acatech, 2016a, p. 64) in increasingly complex technical processes. Moreover, the solution to problems of worker qualification associated with these new processes also seemed readily apparent: the increased use of on-the-job training using digital teaching tools, such as digital media, blended learning, or distance learning (Spath et al., 2013, p. 126).

The debate in the social sciences about the effects of Industry 4.0 on work, competence requirements, and qualification opportunities did not give much credence to this envisaged scenario^1^. For a long time, the social science debate was characterized instead by far-reaching prognostications regarding the future of work without much empirical evidence. As a result, the positions in the debate could hardly be more divergent. Meanwhile, however, the picture for Germany has changed. An increasing number of contributions are based on empirical studies, although there is still a discrepancy between the scope of the debate and its empirical foundations. The present contribution takes up some aspects of this ongoing debate. On the basis of the author's own extensive case study–based empirical work, an attempt is made to clarify a number of open questions related to industrial work and continuing training.

The first question concerns the nature of the change in industrial work caused by the recent wave of digitalization. Often, radical change is assumed. The scope of predicted changes in terms of job tasks as well as required employee competences varies widely. Promoters of Industry 4.0, for example, foresee a large-scale change (“general upskilling”) in the qualification requirements of the whole shop-floor workforce (Acatech, 2016b). Others expect a general downgrading instead (Butollo et al., 2017). Another thesis is that there will be a massive restructuring of companies' employment structure (“reskilling”) such that existing activities (Dengler and Matthes, 2015, 2019) or professions (Wolter et al., 2016) will disappear and completely new ones will be created.

The second question is directly connected to the first and emerges from debates about the needs and opportunities related to firm-based continuing vocational training in the context of digitized industrial work. The focus here is 2-fold: on the need for teaching new competences to workers on the one hand, and on new possibilities for on-the-job learning in the digital work environment and for teaching via digital media on the other. With regard to teaching needs, there is the view that digitalization has increased the need for worker training and that this need must be met above all through new forms of learning at work (Kagermann et al., 2013; Spath et al., 2013) even as the limitations of on-the-job learning have been noted (Dehnbostel, 2019). With regard to new learning opportunities, the authors argue almost without exception that opportunities for competence development have expanded because of digitized work (Dehnbostel, 2019) and digital media (e.g., Ovtcharova et al., 2015; Stich et al., 2015).

## Methods and Empirical Basis of the Study

The DIGIND^2^ project was a multistage study focused on the leading German branches of Industry 4.0. Its aim was to make an initial assessment of the effects of digitalization on work, competence requirements, and continuing vocational training in industry. The core of the study consisted of 10 company case studies in four sectors (the automotive industry, mechanical and plant engineering, the electrical industry, and the chemical/pharmaceutical industry).

Originally, these company studies were intended only as an examination of applications of Industry 4.0 in which the so-called Cyber Physical Production Systems^3^ (CPPS) are used. Before the field work began, however, it became clear that such systems often promise more than they can deliver in operational reality. In terms of production technology, “digitalization” in the context of Industry 4.0 describes a new wave of information-technology penetration, networking, and automation of modern production and industrial support services. The start and development of this wave cannot be dated with historical accuracy (Butollo et al., 2017), and its content cannot be precisely defined. The range of digital innovations begins with the use of new information-technology tools in production processes (e.g., tablets to control a machine, data glasses in logistics). It also includes automation technologies that completely or partially replace certain human tasks and functions (e.g., robots) and new, complex algorithms for controlling production processes. Finally, integrated systems for meticulous recording, planning, and (remote) control of production and service processes are also included, whether in the company's own operations or at customers' premises. A systematic distinction can be made between (mobile or stationary) assistance systems for manual work (Niehaus, 2017) on the one hand, and automation solutions on the other. The latter can be differentiated internally according to whether the actual manufacturing process or its planning and control is to be connected and automated.

The selection of the company case studies was designed to capture variation on three factors: industrial sectors, the form of digitalization interventions, and the core characteristics of shop-floor work functions or activities. This third factor may account for differences in the work-related effects of technology (Baethge-Kinsky et al., 2018). Table 1 gives an overview of the sample of the 10 case studies. It distinguishes between the following three types of digitalization.

**Table 1:** Sample of company case studies.

**Digitization type (number of cases)**	**Description use Case**	**Industry sector**	**Case identifier**
Digital assistance systems for direct and indirect production work (*N*= 4)	“Digital operator Guidance” in mass production assembly	A	A 1
	“Digital operator Guidance” in mass production assembly	E	E 1
	“Digital operator Guidance” in mass production assembly	MA	MA 1
	“Smart” Digital assistance system of maintenance work	A	A 2
Digital automation/autonomization of production (*N* = 4)	Digital integration and visualization of automated assembly and testing	MA	MA 2
	New level flexible automation production	E	E 2
	Automation of process steps and modules	CP	CP 1
	Advanced process control algorithms	CP	CP 2
Digital integration of business processes (*N* = 2)	Target: Automation of planning and control processes	MA	MA 3
	Target: Automation of planning and control processes	MA	MA 4

*A, automotive and automotive supply industry; E, electrical industry; ME, mechanical engineering; CP, chemical/pharmaceutical industry*.

Type 1 represents digital assistance systems for direct and indirect manual work in production. With one exception, these four cases are systems for digital worker guidance in manual assembly. The exceptional case (A2) is an assistance system for maintenance. Type 2 includes four cases of *digitalization as a new stage of automation or autonomization of production*. They are primarily concerned with extended algorithmization in the sense of using complex rule-based controls for existing automated and semi-automated production processes. Two further cases represent *digitalization as digital integration of business processes* (Type 3). Both cases pursue the goal of automating planning and control processes; figuratively speaking, they are solutions for industrial production in which all further planning, control, and manufacturing processes automatically interlock after the order has been placed.

Six of the 10 cases involve production with a genuinely skilled workforce (*Facharbeiter*^4^). In two cases, mixed teams of skilled and semiskilled workers are used. In the last two cases, only semiskilled workers are used (typical also for non-digitalizing firms), and these two cases both fall within the first type of digitalization (digital assistance systems).

The focus of the case studies in factories was the direct effects of digitalization on work, on worker competence, and on qualification opportunities for the affected workforce. Following earlier SOFI studies, we differentiated the types of work to be analyzed by function (production or maintenance) and focus (Schumann et al., 1995, p. 114). To obtain reliable results, we designed the case studies as multiperspective, in-depth research (“cross examination”)^5^. They were conducted through workplace observation and interviews with experts from management, works councils, and employees (see Table 2). The central analytical instrument for the case studies was the job analysis of the affected type of work. For this, different information sources about work and qualification opportunities were integrated. This job analysis forms the basis of the results reported here, and they are discussed from two perspectives. First, they will be addressed in terms of change related to present cases of similar non-digitized work. Second, the effects on work and competence will be compared to the results from studies conducted at SOFI in the 1980s and the 1990s. In this way, we can judge how strong current changes are and whether they are actually attributable to the latest wave of digitalization.

**Table 2:** Instruments used in case studies.

	**Instrument**		
	**Interviews company experts (*N* = 55)**	**Workplace observations (*N* = 15) (type of analyzed work)**	**Interviews affected employees (*N* = 40)**
Duration	Circa 1.5 h	Between 4 and 11 h	Circa 1 h
Participants	Management and specialist staff production	Manual work (production)	Assemblers
	Head of training and further education, personnel development	Manual work (maintenance)	Maintenance men (electrician)
	Management and specialist staff process control engineering	Manual work on machines (production)	Machine operators
	Head of personnel management	System regulation (production)	Electronics technicians for devices and systems
	Works council		Chemistry skilled worker
			Metal cutting skilled worker
Issues/Topics	Concepts	Work situation	Experiences
	Structures	Work procedures	Estimations
	Processes	Work requirements	Valuations
	Experiences	Learning opportunities	Expectations/Wishes
	Perspectives		

With one exception, all of these case studies were conducted in 2017. In each case, we were able to implement the entire survey program as planned. With a few exceptions, all conversations were recorded and then transcribed and made anonymous. The results of each case study were immediately evaluated and reported back to the companies to check our interpretation. The discussion and analysis documents prepared in each case, as well as other accessible company documents, were evaluated both within and across cases.

## Main Results

### Effects on Work and Competence Requirements

#### Job Task Profiles: New Accents, Not Radical Change

The German history of work in industrial production was and still is a history of the incremental transformation of tasks, performance conditions, and competence requirements caused by the design of technology and organization. New types of work with characteristics affecting specific job profiles have always emerged without completely replacing conventional types of work. The simultaneous coexistence of different types of work in one and the same company (e.g., handcrafting, assembly-line production, and the regulation of automated production systems) is evidence of this fact. It is further demonstrated by differences between industrial sectors in terms of the technical and organizational pace of modernization and of associated work and task structures (cf. Kern and Schumann, 1977, 1984; Schumann et al., 1995). Inspired above all by international labor market and technology studies (cf. Frey and Osborne, 2013; Brynjolfsson and McAfee, 2014), national forecasts with a strong technical focus (cf. Dengler and Matthes, 2015, 2019; Wolter et al., 2016) often prognosticate a major restructuring of work. The thesis is that digitalization should make the typical tasks of skilled and semiskilled industrial labor disappear because these tasks can be automated. Following this line of argument, traditional activity profiles of production work should completely disappear or lose elements that were previously essential.

Across all analyzed cases, our findings show that the digitalization of industrial production has not changed and likely will not change work as radically as originally predicted, for two reasons. First, in only one case did a firm make significant changes in its approach to the organization of productive work. This applies to the vertical and horizontal division of labor as well as to the deployment of the workforce. Attempts at change were either abandoned (ME3, ME4) or involved merely the official adoption of already existing, informal division-of-labor practices (ME2). Second, digitalization solutions were used mainly in support of work, not to control it. This applies to the two cases involving digital assistance systems for manual work as well as to the six cases using higher degrees of automation and integration. As discussed below, this does not mean that management did not have plans and ideas about using digital technologies to replace human interventions. However, after the imperfections in digitalization technology were exposed, management often left the final decision about how to use it up to the employees. The fact that current forms of digitalization do not change, on their own, the type of work in which they are applied becomes clear when one examines the objects to which the affected work tasks are directed.

Note, for example, the systems for digital worker guidance in manual assembly. There, a product to be assembled is navigated through the different stations of an assembly line using an RFID chip^6^. The entire assembly process is stored in detailed work steps in system control. All these steps are displayed to the assemblers at the respective station on a monitor in the form of written instructions and/or visual representations (photos or video sequences). The completion of the individual work steps is either automatically checked (e.g., via integrated image processing systems) or manually acknowledged by the workers (A1, E1, ME1). In one case (ME1), the digital worker guidance was combined with a “pick to light” system for selecting the correct assembly part. A kind of traffic light system indicates which part is to be installed (green) and which part not (red). Another case (A2) involved an assistance system for maintenance, by which fault reports and maintenance orders are transmitted to production and accepted by maintenance personnel. The system contains features, such as spare parts reservations, system plans, and documentation on the fault history, all of which can be accessed via a smartphone or a tablet.

In no case of digital assistance systems for manual work does the type of work itself changes. This applies to manual assembly work in the three cases with digital worker guidance as well as to manual maintenance work in the case of the maintenance assistance system. Manual assembly work in series production using worker guidance systems has the same characteristic features of semiskilled work without them. These include, for example, a high degree of monotony in the work, the need to tolerate routine activities, and the scarcity of opportunities for technically demanding, problem-solving action. However, the scope of human action in work processes varies between cases, depending on the density of the digital monitoring and the control network. On the extreme end of the spectrum is the case of the automotive supplier (A1) with comparatively long cycles of assembly (~20–30 min) and long times between the automatic control procedures. The other pole is represented by the case of ME1 with its short cycles of assembly and almost permanently running control procedures.

The job profile of the maintenance man (A2) remains that of a skilled craftsman. His work is often characterized by time-consuming repairs of machines or plants, for which, for example, drives or other aggregates have failed or no longer function with the desired reliability. The assistance system reduces waiting times and walking distances (e.g., between maintenance workshop and warehouse). But this does not change anything about the range of tasks that have to be performed. Considerable shifts in the amount of time allocated for tasks and in the specific content of tasks are observable, but these apply to a task spectrum that has remained stable. However, this has less to do with the use of digital assistance systems than with the fact that the work task itself has changed. The operational expansion of the “preventive maintenance” concept increases the number of planned interventions as well as time spent systematically analyzing weak points. This means that the situation at work is increasingly characterized by other tasks than the execution of unplanned manual repairs.

Also for the four cases of *digital automation* (Type 2), we do not find any fundamental changes in job profiles induced by the new digitalization technologies. As a rule, the traditional task profiles of skilled system regulators and semiskilled personnel at the machines and equipment remain largely unchanged. Digitalization does not completely eliminate any previous core tasks, nor does it add any completely new tasks. System regulators remain entrusted with the control, regulation, and optimization of machines and processes. And they intervene—as far as possible with foresight—whenever technical malfunctions threaten or actually occur. Insofar as qualified and semiskilled personnel are deployed together, semiskilled machine operators continue to focus on tasks, such as material feeding, loading equipment, or random checks of finished parts. In these cases, digitalization is primarily used as a medium for faster transmission and more comprehensive collection and presentation of relevant process information. The personnel must react to this when carrying out their own work order. There has not been a single case of digital automation in which the leaps in technology associated with digitalization have had a direct impact on blue-collar work tasks. Here, one must keep in mind that in all these cases, digitalization is not completely new. Rather, it is an augmentation or updating of already-existing digital production technologies and, accordingly, of the type of skilled and semiskilled automation work involved. Digital technologies are primarily concerned with extended algorithmization in the sense of using complex rule-based controls for existing automated and semi-automated production processes. The case ME2 stands for the digital networking and visualization of the subprocesses of assembly and testing within a flexibly automated production of circuit boards. Here, process states, results of automated test operations, and malfunctions are displayed to the operating personnel on control monitors in real time. They are used by them for control corrections, such as mass data-based optimization of the manufacturing programs. The case from the electrical industry (E2) represents a new level of flexible production automation. There, the unit to be assembled navigates independently through the individual stations of a fully automated assembly line. It only docks at those stations where an assembly step actually has to be carried out. One of the two cases from the chemical-pharmaceutical sector represents the discontinuous production^7^ of different chemical specialties (CP1). For a long time already, production there has been monitored and controlled by a digital process control system. The innovation for the case study involves an IT-based automation of step chains during the “start-up” of plant components and of individual process steps (e.g., distillation). The other case (CP2) involves the continuous production^8^ of a single product. In the past, production already had been monitored and controlled by a digital process control system; the digitalization innovation here involves the extended algorithmization of process control (advanced process control) via a large number of complex software programs that control and regulate individual subprocesses in reference to relevant parameters.

The cases of *digital integration of business processes* (Type 3) can serve as a litmus test for a radical change of job profiles. Both cases (ME3, ME4) correspond most closely to the vision of a cyber-physical production system. In these cases, all planning, control, and manufacturing processes automatically interlock after the order has been placed. In one of the two cases (ME3), this approach has been implemented to such an extent that all planning and control processes are automatically triggered and executed by ordering the product on the internet (e-shop). The production order is transferred within 15 min to the control system of the production cells and processed on the machines according to the “first in-first out” principle. In the second case (ME4), the order is still entered manually, but then the process continues as in the first case, albeit with two main differences. First, the machining programs are generated automatically via a CAD/CAM interface. This interface is coupled with a learning software package. It registers exactly to what extent and how often certain program data are corrected by the machine personnel. It takes this into account when programming subsequent orders. Second, the machines are equipped with a sensor system for detecting running noises and a coupled software package. It can detect irregularities and, if necessary, transmit corresponding fault messages to a “smart watch” of the operating personnel.

In both cases—the only ones from our sample—management initially assumed that the innovations would bring significant change in the task profiles of the skilled workers traditionally employed there. The assumption was that two of the previous core tasks—the optimization of machining programs and monitoring of running processes—would be practically eliminated. The following passages are revealing.

So the employee standing there at the machine no longer needs this programming know-how. The software takes care of that (Manager Production area ME3-1).

Because the system [of programming and monitoring] does it. I don't need anyone else with the experience. I actually only need one more person who, together with the system, clearly defines the processes once. Who then prepares the fixtures. And for everything else I don't need any skilled worker (General Manager ME4-1).

Our observations of the workplace show that these expectations did not bear out in reality. In the case of the skilled system regulators employed in these firms, digitalization does not completely eliminate any of the tasks they mentioned having done before (these included optimization of programs, monitoring of running processes, and plant loading). In the case of ME3, this is due to the fact that, among other things, those orders continue to be entered manually into the process for which no automatic production programs can be generated, and this task falls to the skilled workers. In the case of ME4, the automatically generated machining programs are considered to be very reliable. However, they are economically suboptimal (long machining times), especially for large quantities, and are therefore optimized accordingly by the machine personnel. What changes in these cases is the frequency with which programs have to be written for largely new parts. This frequency decreases as well as the frequency with which the clamping of the workpieces on the machines must be planned. Every program run, every clamping operation is stored and documented digitally in the form of data and images, so that it can be accessed when production is repeated. Nevertheless, in both cases, this means that the time spent on programming tends to decrease significantly. On the other hand, the tasks of system monitoring and loading as well as the quality control of finished workpieces take up more time. In the case of ME4, the automatic transmission of error messages to the “smart watch” of the operating personnel ultimately does not make their presence on the machine superfluous. This is because the reporting threshold is set relatively inexactly and messages are not sent continuously. As a result, the skilled worker observes the ongoing production process more frequently than planned.

In all cases examined, digitalization in industrial production has not yet led to radical changes in the task structure of skilled or semiskilled work. Rather, job profile changes are characterized by considerable shifts in time and content all within a stable task spectrum.

#### Upskilling, Downgrading, or Reskilling?

There is still much controversy about the extent and direction of the changes that new digitalization technologies and, in particular, the use of artificial intelligence might bring for the skills required for industrial production work. The current debate is characterized by different predictions about the direction that the qualification structure as a whole will take. The scenarios vary between “general upskilling” (cf. Acatech, 2016b), “downgrading” (cf. Butollo et al., 2017), and “reskilling” (cf. Wolter et al., 2016). In connection with this, there is a discussion about the required competence profiles of industrial work. Emphasis is placed on the fact that digitalization leads to new profiles of skilled workers in which IT skills are added to (unspecified) technical skills. In addition to this, cross-disciplinary competences especially seem to be gaining in importance (cf. Zinke, 2019). All in all, the predictions make it seem as if technical skills required in the past will become superfluous, replaced by new skills (reskilling). Following this argument, we should see changes in the competence requirements of digital production work involving new digitalization technologies and, in particular, the use of artificial intelligence. Moreover, these changes must be related to the recent wave of digitalization.

##### Downgrading as an Exception

There is only one case in our sample where digitalization changed the competence requirements of skilled or semiskilled work in the sense of downgrading. We find this exception in the case of ME1 with its short cycles of assembling and almost permanently running control procedures. Here, the use of digital worker guidance minimized the scope of worker autonomy to such an extent that the semiskilled assembly worker can mentally “switch off” while working.

Well, you don't have to think anymore. You can actually stop thinking when you're working, because you're simply intervening where the light comes on. (…) So I couldn't do that for the rest of my life, because then I'd go stupid (Assembly worker ME1-1).

When we presented this result to the management, a discussion began there about whether the rigidity of digital control should be reduced.

##### More Upskilling Than Reskilling

As mentioned above (see the section Job Task Profiles: New Accents, Not Radical Change), only minor changes are observable in the digital-work job profiles analyzed. Above all, changes can be seen in the relative amount of time different tasks take and in the object of those tasks. Thus, on the one hand, certain professional skills previously required to complete the tasks remain relevant. On the other hand, the same task requires both new technical and cross-curricular competences, because its object has changed. In the cases of digital automation (Type 2), for example, the system regulators are still responsible for the smooth running of the system. Especially in these cases, the technical qualifications that were necessary previously have to be partially supplemented by IT knowledge. This is because some faults can only be rectified by intervening in the control system of the plant. Another example is the optimization of production processes. This was a typical task of skilled automation work before the recent wave of digitalization (cf. Schumann et al., 1994). On the one hand, this task is associated with theoretical and empirical knowledge of the respective production and processing methods as well as of plant engineering (electrics and mechanics). Now optimization needs knowledge about the IT-based functions of the plant engineering and the data they produce. On the other hand, the task is associated with methodological-analytical competences (problem-solving skills). However, the problem-solving skills required in digitized processes appear to require different conceptual foundations, such as the understanding of algorithms (digital competences).

Still, these changes represent less a radical than a gradual change in competence requirements. This can be seen both in the importance of technical qualifications and in the importance of cross-curricular competences.

##### Technical competence: augmentation, not replacement

Change in technical competence has been moderate because traditional competences have not become superfluous. On the one hand, this concerns *requirements for manual skills or manual dexterity*. These have always characterized manual activities in industrial production in particular, but workers still need their hands even when digital assistance systems are used. In all cases with digital worker guidance (A1, E1, and ME1), manual assembly operations still require considerable manual skills. This applies for example in the case where small parts are to be installed in places that are difficult to access. The same applies to the maintenance man in case A2, when repairing a defective engine on a machine or system. And even in the context of work in automated or connected production (cases ME3 and ME4), a certain dexterity (e.g., in the handling of tools to rectify faults or when clamping workpieces) still remains significant.

On the other hand, *technical knowledge* plays a role in making change gradual. Digitalization solutions do not automatically render specialized workforce knowledge used in previous production and work processes superfluous. With only one exception (case ME1), theoretical and practical product knowledge is a prerequisite for the execution of assembly operations at all times. This means, e.g., knowledge about the design of a unit to be assembled or about the consequences of assembly errors for the functionality of the final product. Automated and connected production also requires theoretical and empirical knowledge of the respective production and processing methods as well as of plant engineering (electrics and mechanics). This knowledge does not become less important, but rather remains an important basis of successful work. This applies to the assessment of process states and faults as well as the initiation of process corrections and suitable measures for fault rectification. In two cases (ME3 and ME4), the importance of this type of professionally structured knowledge (experience) was initially underestimated. They deployed semiskilled workers who repeatedly found themselves in overly demanding situations. Afterward, this misjudgment was corrected by the introduction of qualification measures or by changes in personnel deployment.

Both the depth of technical knowledge required and the relevant knowledge domains differ on the following dimensions of the production context.

- *Complexity of production processes*. Production processes differ considerably in the number and structure of the parameters that influence the subsequent production result. Highly complex processes include the distillation and drying processes in the chemical industry and the turning and milling of workpieces. Less complex processes include the mechanical punching of sheet metal or the grinding of metal. Depending on the complexity of the processes, distinctive process-specific knowledge forms an essential core of technical knowledge. In our sample, this applies above all to both chemical industry cases, but also to the case ME4, in which workpieces are turned and milled on CNC machining centers.
- *Level of mechanization*. At a higher level of technology, the knowledge relating exclusively to the process (e.g., assembly, machining, and distillation) is no longer sufficient to guarantee trouble-free production. Here, knowledge of machine and plant engineering, i.e., on its mechanical, electrical-electronic, and information technology components and their functions, is of increased importance. It also provides the basis for fast and reliable fault detection and subsequent interventions. Particularly in cases of automated and connected production (E2, ME2, and ME3), knowledge of the information technology used is becoming increasingly important. This is because malfunctions are more frequently associated with software failure. Some causes can only be identified by viewing the source files of the programs or can only be remedied by corrections in the programs.
- *Work organization*. Depending on the extent to which responsibility for the smooth running of the production processes is hierarchically and functionally divided, the depth of expertise required from automation workers in the respective areas also varies. For example, where a separate maintenance department is exclusively responsible for the functioning of the plant technology (as in CP1 and CP2), the skilled chemical plant workers themselves only need basic knowledge of the plant technology used. The situation is different in cases E2 and ME3. There, the system regulators are considered responsible for plant technology including its maintenance.
- *Type of production*. There are differences in the frequency with which production or product parts with new design features, properties, and dimensions are manufactured. This frequency is a key factor in determining the extent to which process-specific knowledge must be available close to the production process. Here it is true that a unique manufacturer, such as ME4, who rarely produces a largely identical or similar machine, requires a high level of process-specific knowledge on site. Conversely, this does not apply to mass production, as in cases A1, E1, and ME1, where process development is completed before handover to production.
- *Innovation intensity*. The speed at which a product is or must be launched on the market is a factor determining the knowledge needed on the shop floor. For a long time, the chemical industry was regarded as a prime example of science-based production. There, in-depth process-specific knowledge was required above all from the chemists and engineers. They developed the process and translated it into a production specification. This specification contained detailed instructions for the use of substances in apparatus or process parameters to be observed (e.g., temperature), which the workers had to follow. In the case of CP1, this sequence of planning and implementation steps can no longer be adhered to. Meanwhile up to 20 new products are now produced each year, for which there may be incomplete process instructions. In this respect, process knowledge is becoming more, not less, important for workers in the chemical/pharmaceutical industry.

*Cross-curricular competencies: not completely new, just newly accentuated*. A special feature of work in automated and connected production seems to be the *need for a corresponding ability to interpret data*. In order to recognize that a process is getting out of control, the worker repeatedly has to establish references between available digital information about process states and real process behavior. This requirement was recognizable in all automation and connecting cases. In work processes, this need is reflected above all in the fact that the system regulators, such as in the case of chemical continuous production (CP2)—individually compile the set of process parameters on their observation monitors that in their experience is best suited for tracking a particular production process.

Digitized work is almost universally combined with *needs for communication and organizational competences*. As practically all cases show, work in digitized industrial production is integrated into technically and organizationally more closely interlinked processes. For example, to ensure that various subprocesses can run synchronously, it is necessary that colleagues permanently communicate, coordinate, and support each other. In the case of the CP2 chemical plant, for example, the system regulators in the control room have to communicate with colleagues who have to connect and disconnect plant components on site. In addition, there are requirements for organizational interpretation and decision-making skills as well as self-organization skills. This is generally the case if, contrary to the production plan, an ongoing process (ME4) or a certain production step in batch production (CP1) takes longer and blocks production capacities. Increasing demands on communicative sensitivity can also be recognized. It results from the fact that a large number of technical and organizational disturbances need synchronous processing and mastering of production orders. The demands on interdisciplinarity (cooperation with unfamiliar specialists) should not go unmentioned; especially in the highly complex systems, it is always necessary to exchange information with differently or more highly qualified personnel.

Recent literature offers no new findings about the need for cross-curricular competences in digitalizing environments. Our results largely confirm the already established findings of the occupational sociology qualification research of the 1980s and the 1990s. In those decades, research emphasized the importance of the above-mentioned aspects of cross-curricular competences (cf. Schumann et al., 1994; Baethge and Baethge-Kinsky, 1998, 2006). We assume that the stronger networking effect generated by digitalization does not lead to completely new cross-curricular competences but that it accentuates them more strongly than in the past.

However, we see an exception to this in some cases (ME2 and CP1) of digitized production. In these cases, the skilled automation worker took over tasks of a systematic process improvement on the basis of new mass process and plant data. Problem-solving competence in the sense of a structured approach to problem analyses, a pronounced ability to interpret data, and abstraction capability were generally required of these employees for their process optimization work even before the current wave of digitalization. However, this was primarily achieved by drawing on the experience they had gained in their previous working lives with a specific process or plant. According to the observations and discussions from the case studies, the problem-solving competence required in dealing with “big data” is different. It seems to require a different knowledge base than that is typically included in dual initial entry-level training and subsequently further developed in professional practice.

I worked outside in production for 10 years (as a skilled chemical worker) but didn't know the theory. So, and many theorists or engineers or academics, they don't know production. They have super great systems, but sometimes they don't know the crux because they didn't work outside in production. I just enjoy the advantage that I slowly have both (System Regulator CP1-1).

In this self-description, a skilled worker refers to the study of chemical engineering that he took up about a year earlier. In the search for automation solutions (algorithms), he now was able to take a different, more theoretical look at the analysis of the production processes and the process data that arise there.

Although no other worker in our cases who took on such complex optimization tasks so clearly states the advantage they perceive from gaining basic theoretical training in their field, this career path is by no means an exception for people who take on such tasks. Rather, a combination of initial training, vocational practice, and continuing vocational training is a prerequisite for such task profiles in other cases. These tasks are often voluntarily taken on and successfully mastered by persons who have also begun or completed training as technicians or as foremen (“Meister”^9^) or by persons with degrees from universities of applied sciences. Conversely, skilled workers who have not completed additional further training may have clear reservations about taking on such tasks.

#### Continuing Vocational Training: Intensified, On-the-Job, and Based on Digital Media?

With the start of the latest wave of digitalization, a debate began over intensified continuing vocational training, including what content is meaningful and what forms and formats are appropriate. The tenor of the debate can be summed up as follows. Above all, digitalization requires an increase in company training efforts. Continuing education must be organized close to the work process (close to or directly in the workplace) and should in particular make use of the learning potential of digital media (Kagermann et al., 2013; Spath et al., 2013; Ovtcharova et al., 2015; Stich et al., 2015). Specific attention is given to the opportunities and risks of learning close to work (Dehnbostel, 2019).

If these arguments hold, significant changes in firm-based training must be observable. First of all there, we should see intensified training on and off the job. Second, more learning opportunities close to the places where digital work has to be done should be in evidence. Third, we should be seeing the greater use of digital media. However, our results tend to show a quite different picture.

##### Further Training in Enterprises: No Major Increase

First of all, we did not observe any intensified training in connection with digital work that aims at more than a superficial understanding of digital technologies and their functions (“being able to operate”). The reason for this is that changes in work requirements often remain undetected and thus never become the subject of continuing vocational training. Usually only the immediate demands on workers resulting directly from the interaction with the respective digital technology and its handling are detected.

Accordingly, in most cases, workers were instructed in the use of digital technology within the framework of continuing education. Only in two cases (E2, ME2) had the development of a planned continuing vocational training concept not yet been completed. In another case, in which continuing training was declared by company management to be an individual obligation (ME4), the “training” of the skilled workers employed there was carried out on the job through discussions with colleagues in the planning department. In the seven other cases examined, further training courses tailored to the respective digitalization use case were offered. In most cases, instruction focused on practical application and did not involve a deeper understanding of the technology.

In one of the cases with digital worker guidance in assembly (A1), training lasted several weeks and consisted of two parts. One part was the detailed training about the product to be assembled and the dangers connected with its assembly (high voltage). The other part was on-the-job training using the pictures and videos of the individual assembly steps stored in the control system of the assembly plant. In the remaining two assembly cases with digital worker guidance (E2, ME1), the master instructor gave brief instructions at the workplace. There the pictures, videos, and written instructions for the individual assembly steps were also used. In the case of the intelligent assistance system in maintenance (A2), a training course lasting several hours was carried out in which the essential functions and features of the system and the handling of the user interface were first explained. Then the participants were able to try out the features.

In the case of the specialty manufacturer CP1, training related to digitalization technology has been made a permanent part of an introduction program designed for new employees. The program combines off-the-job training on the process control system with a mentoring system in which an experienced colleague takes over the supervision of the new colleague on the job for 1 year. In the other two cases of automated or connected production (CP2, ME3), the technology-related qualification consisted exclusively of rather unsystematic on-the-job training. It was part of a mentoring system in which colleagues or superiors familiar with digitalization are available to impart knowledge about the handling of the technology.

The cases of in-company continuing vocational training courses described above appear at first glance to be appropriate. In reality, however, they are usually limited to the outwardly visible changes in how the new digital technology is handled, leaving less visible changes often undiscovered. This applies to those requirements that result primarily from general work responsibility but are not included in the operational task descriptions. If such requirements are not covered by existing competences, they manifest themselves in excessive demands and permanent stress of the workers. Stress reactions of this kind were observed in several cases; in some instances, this was the reason for company to participate in our study. One reason above all led to problems: a lack of systematic operational analysis of the changes in digital work. Indeed, digitalization cannot be said to have been well-received in our cases if understood as an issue that needs to be anchored both organizationally and in terms of the content of in-company continuing vocational training. This is a problem of personnel and training that has been adequately dealt with in very few cases. In only two cases (ME1, E2) did the company HR department carry out an initial assessment of qualification requirements; the basis for this was formed primarily by expert surveys in the application areas of new digitalization solutions, in one case combined with an employee survey.

##### Learning at Digital Work: Fewer, not More Opportunities for Competence Development

The analysis of our results shows that digitized industrial work allows for less learning at work. This is because of a growing tension between specific needs for and conditions of competence development at work. The reduction in learning opportunities concerns first and foremost learning at work in general. In particular, the acquisition of experiential knowledge that is indispensable for coping with work tasks is becoming increasingly difficult. Learning at work is going to be more difficult because the conditions of work (disposable time, opportunities for acquisition of experiential knowledge) are changing.

In terms of disposable time, only in the case of manual work is there any significant amount of time for the conscious use of learning opportunities. The situation is completely different in the cases of digital automated or connected production (Types 2 and 3). There, automation workers must give their permanent attention to the production process and have no time to learn anything new.

In the automation and connection cases (Types 2 and 3), it is almost always possible to identify a threat to the way that employees sharpen their skills themselves through the work process. This form of self-teaching has been important for the development of competence in industrial work in the past. There are various reasons why it is threatened. One is that the frequency of necessary interventions in programs and processes decreases due to more stable and technically more secure processes. This can be observed, for example, in the case of the chemical plant CP1, where the new control algorithms ensure more stable processes overall. The higher overall stability of the production processes diminishes the learning opportunities that arise from coping with difficult process situations. There is another situation we find in the case of ME4. Here, the “learning” CNC programming software ensures that employees make fewer and fewer corrections because the system basically imitates their programming style. Last but not least, the encapsulation of systems lowers sensory access (hearing, seeing, smelling) to processes, and sensory information has always been an important source of experiential knowledge.

A much more differentiated picture emerges for digital assistance systems for manual work (Type 1). There, learning opportunities depend on how tight the network of digital control and steering of the work process is. Only in the case of close monitoring are there no possibilities of acquiring empirical knowledge. In such cases of “low-learning work,” the motivation to learn or the willingness to take responsibility is undermined. The following case of an employee on an assembly line (ME1) equipped with digital workers' guidance can be regarded as exemplary. The employee placed the completely assembled aggregates in a box at the end of the line and was made aware by a colleague who happened to be passing by that one of the assembled aggregates was missing. His answer, that “[t]his can't be, the computer tells me that everything is all right” (Assembler ME1-3), shows not only that he has become accustomed to the technical assistance system guiding him through the process but also that in such contexts, personal responsibility and autonomous thinking can fall by the wayside.

The declining opportunities for learning at work associated with the specific case of digitalization generally remained unrecognized within the firms for a long time. This has two main reasons. The first reason is that the management does not or cannot recognize the indirect effects of decreased learning opportunities at work, such as excessive demands on workers, illness, or errors. The other reason is that the conditions under which learning can take place are often not sufficiently reflected upon, especially by production management. An example of this is the suggestion made by the plant manager in the CP2 case. In response to finding that learning on the job was not possible in day-to-day business, he made the following suggestion:

If that is the case, why don't we just move the qualification to the end of the [12 h] night shift? (Plant Manager CP2-1)

How much attention employees can still muster at the end of a 12-h shift for successful learning processes was not an issue for him. The reasons for the underestimated problems of learning at work lie above all in one fact. It is not understood that digitalization of work leads to declining opportunities for learning at work if no active countermeasures are taken.

##### Limited Use of Digital Media for Firm-Based Continuing Training

Further training in firms based on digital media is a learning format that—judging by our case studies—so far has been of only limited use. Digitalization as an explicit subject of continuing vocational training in enterprises played practically no notable role in any of the cases we investigated. Moreover, there were no didactically structured digital learning opportunities directly accessible to employees in production. The reasons for this gap are organizational, financial, and cultural.

From an organizational perspective, digital technology is conceived of first and foremost as an object of work, not of learning. Currently, digital technology plays an important role in learning only in cases of assistance systems for manual work. It is used for demonstration and practical comprehension of skills within the framework of initial training or for refreshing knowledge. Within the assistance system for maintenance as well as in the automation and networking cases, monitors and mobile terminals are used for illustrating presented information and to let workers try out features. The use of digital learning media therefore played a role in our concrete cases of digitalization, albeit it was quite limited in the automation and connection cases in particular. In both cases from the chemical/pharmaceutical industry (CP1 and CP2), the use of simulation software is under concrete consideration.

From a financial perspective, the use of digital media for firm-based continuing vocational training seems to be limited by the cost of developing or purchasing such media. This applies especially to the use of complex simulation software for highly automated and/or connected production. The costs of such software:

…quickly reach the range of one million euros and more. This only really pays off if you have a whole series of identical production plants. Then the costs can be distributed (General Manager CP2-2)

In addition to pure cost aspects, the question of potential benefits also plays a role. In the presentation of our findings in the two chemical cases, limits to the acquisition of knowledge through digital technology were a topic of discussion. It was also discussed to what extent one could switch over to a non-automatic, “manual mode of operation” for teaching purposes. In the view of the management, this may be a better way to give employees the kind experience their older colleagues have.

From a cultural perspective, it cannot be taken for granted that companies will be eager to use digital learning media. Especially production management has to learn, for example, that

…the search for information or suitable learning programs must be understood as “learning at work”—and not as a break from work (Head of Personnel Development ME3-1).

## Conclusion and Discussion

The German debate on the effects of the recent wave of digitalization on work and further training in industrial production is still characterized by two main points. One concerns the question of how fundamental the expected changes to work and competence requirements really are. The other point touches on the need for continuing vocational training and the opportunities for developing competences on the job or in proximity to it. The last point includes the questions about the role of digital media.

As can be seen from the results of our case studies in newly digitized production and maintenance processes, there is no evidence of radical change in the labor process or in work demands. For the most part, previously existing task profiles of skilled, semiskilled, manual, and automated work are unchanged. However, within this stable task spectrum, considerable changes in the amount of time allocated for tasks and in the specific content of tasks are observable. Connected with this, we observe moderate changes in competence requirements in the sense of a minor upgrade. This means, first, that traditional professional skills, based to a large extent on experience, have not become superfluous and are not likely to become so. Second, cross-disciplinary competences, such as communication and cooperation skills, abstraction capabilities, or problem-solving skills will be even more important than in the past.

Some new competence requirements are emerging, however. The task of process optimization is indeed one area that clearly requires new competences. It requires new technical knowledge about IT-based plant engineering and the data it produces as well as new IT-related competences like the understanding of algorithms. It also requires the ability to take a theoretical approach to analyzing production processes and to problem solving.

Our finding of rather moderate changes in direct and indirect (i.e., maintenance) production labor in the course of digitalization accords with recent empirical labor research in Germany. The same applies to reasons for why established patterns persist. One reason is that industrial digitalization is generally an incremental process. It is taking place more slowly and cautiously than the promoters of Industry 4.0 had anticipated (Kuhlmann and Voskamp, 2019). Another reason lies in the continuity in the utilization of labor (“path dependency”) as seen in the persistent use of a traditionally functional division of labor as well as in the preference for traditionally schooled workers (Hirsch-Kreinsen, 2018).

Moreover, our analyses of the requirements for qualifications and competences largely concur with current empirical research on two essential points. One point of agreement lies in the emphasis on knowledge of information technology as a new technical core qualification of digitized work; the second congruence lies in the consistent references to the increasing importance of interdisciplinary competences (cf. Zinke et al., 2017; Conein and Schad-Dankwart, 2019; Knieling and Conein, 2019; Zinke, 2019). Relatively new, however, is our finding that today more theory-based problem-solving competences are required to cope with the tasks of optimizing digitized production systems. This competence requires a more science-oriented way thinking and a learning habitus. How well such a habitus can also be developed in traditional dual training as well as in firm-based continuing training is a question for further examination.

Another issue is related to the need for and opportunities of firm-based continuing training. Countering the assumption that digitalization would be associated with intensified firm-based continuing training, increased learning opportunities at work, and the more frequent use of digital media, our findings show a different picture. First, there is a little increase in firm-based continuing training. Training is concentrated only on the most visible requirements for handling innovative digital technology. The less visible changes in work often remained undiscovered and played no notable role in the continuing vocational training courses offered by companies in our cases. Second, the learning opportunities of digital work tend to decrease rather than increase. This reduction in learning opportunities first and foremost concerns learning at work in general. It is only in the cases of manual labor that companies provide any significant amount of time that can be used for the conscious use of learning opportunities. In automated and connected production environments, occasions in which employees can acquire the experience they need to cope with difficult process situations are becoming increasingly rare due to more stable technical processes and to the physical encapsulation of systems. This problem often remains undetected by management. Third, there is limited use of digital media for competence development on and off the job. Digitalization as an explicit subject of continuing vocational training in enterprises plays no notable role. Additionally we could see that there are no didactically structured digital learning opportunities accessible at work by production employees. The reasons for this lie in organizational, financial, and cultural restrictions. In the cases of manual work, digital media are foremost an object of work, not of learning. The costs of digital learning media vary depending on the content and rise massively with the complexity of the material. Last but not least, the use of digital media is still too rarely recognized as a meaningful, integral part of the labor process, especially by production management.

The (at best) moderate increase in continuing vocational training in enterprises and an unenthusiastic use of digital learning media is not surprising. They accord with quantitative research on continuing training (Bundesministerium für Bildung und Forschung, 2019). Especially, the low use of digital media can be explained by the fact that no clearly positive effects of digital learning environments on learning outcomes have been proven yet (Hämäläinen et al., 2015, 2018; Fehling, 2017; Lucignano, 2018). What is surprising here, at least at first glance, are the following two points.

The first point is the often undiscovered tension between experience-based professional competences and the possibilities of acquiring and expanding them in complex, highly connected processes. We have been aware of this problem since learning about “ironies of automation” (Bainbridge, 1983). Nevertheless, awareness of this problem apparently has not found its way into operational technical or organizational concepts. It cannot be ruled out that exaggerated expectations of the performance of digital technology (Lee and Pfeiffer, 2019) in particular plays a significant role here. Above all, however, it highlights the fact that digital work does not automatically improve learning opportunities (Dehnbostel, 2019), but in fact tends to reduce them. The reason for this is the lack of innovative organizational concepts. Concepts are needed that provide a clear framework for the systematic involvement of employees in innovation processes associated with digitalization and in the corresponding learning and development processes. To manage the change to digitized industrial work, a number of organizational measures within the enterprises are also needed, including establishing learning as a central aspect of how work is organized.

The second point is that while the changes in competence requirements may indeed be moderate, they are hardly reflected at all in the continuing vocational training courses offered by enterprises. There are two possible explanations for this. One of them refers to the demand for cross-curricular competences. These competences already were required for skilled industrial work before the new digitalization wave (Baethge and Baethge-Kinsky, 1998, 2006; Baethge, 2018) and have been an explicit subject of entry-level vocational education and training in Germany since the late 1980s. Companies therefore could trust that their skilled workers have these skills to a sufficient extent. The other explanation is related to the technical qualifications and the associated types of problem-solving competences that are required today. These new competences are often undetected by firms. The reason for this is that, in fact, there are enough qualified workers in the labor force able to fulfill optimization tasks in digitized production without additional firm-based continuing training.

This last finding challenges the established concept of skilled work as uniform in terms of qualifications, competences, and above all, how a skilled worker is trained (Meyer and Haunschild, 2017; Meyer, 2018). If qualified work becomes transformed so as to require a more systematic integration of digital optimization competence for all workers in the future, this has above all one central consequence. The access to skilled industrial work will hardly be possible without additional continuing training at a technical college or university, more or less directly after initial entry-level training. Those wanting to stabilize the German system of skilled work have to face the task of incorporating old and new requirements and career perspectives into a binding curriculum of initial vocational education and continuing vocational training. But there is still a long way to go before this occurs in Germany. This is true both for the introduction of digital-relevant competences in continuing education programs at universities (Beutnagel et al., 2018) and for the idea of making entry-level vocational education more knowledge-oriented (Kaßebaum et al., 2016).

## Data Availability Statement

The raw data supporting the conclusions of this article will be made available by the authors, without undue reservation.

## Ethics Statement

At the time the study was carried out, qualitative social science studies in Germany were required to comply with the provisions of the Federal Data Protection Act and the right to informational self-determination of the individual laid down therein. Accordingly, the interviewed participants of the study were informed in advance in writing about the aim of the study, the voluntariness of their own participation, and the principles used to handle the data collected (confidentiality of the information, anonymous results, secure data storage on one site only). They signed a declaration of consent to participate before the beginning of the interview; this declaration also included a reference to a possible revocation of consent at any time with the consequence of the immediate deletion of the collected personal data.

## Author Contributions

This work was the product of collaboration with colleagues from SOFI Göttingen (Kai Marquardsen and Knut Tullius). VB-K developed the design of the study and carried it out. The presented interpretation of the study results is the sole responsibility of the author. Nevertheless, the interpretation of the results was discussed with colleagues at SOFI.

## Conflict of Interest

The author declares that the research was conducted in the absence of any commercial or financial relationships that could be construed as a potential conflict of interest.

## Footnotes

^1^In this paper, the term “competence” describes a quality of individual workers and is preferred to “qualification,” which is more closely associated with job tasks. Note, however, that “qualification” as an indication of externally defined job-performance requirements and “competence” as a term describing individual performance are indeed moving closer together in meaning because job-performance requirements are increasingly based on individuals' situational performance in the perception and interpretation of problems (Baethge, 2011).

^2^The acronym DIGIND stands for the project “Demographic Development, Socio-Economic Structural Change and Digitalization of the World of Work: An Empirical Study on New Forms of Work, Qualification Requirements and Continuing Training (Needs) in Industry.” It was conducted at SOFI Göttingen between 2016 and 2018.

^3^Cyber-physical production systems are production facilities, products, materials, and transport systems that connect intelligent objects on the basis of an “Internet of Things.” These systems autonomously organize and control objects along with the sequence of their manufacturing processes and associated logistics, and they adapt the objects to external requirements such as changing demand and unexpected process disruptions (cf. Bauernhansl et al., 2014).

^4^*Facharbeiter* means a type of occupationally skilled non-academically trained workforce that is specific to the German industry. The competences and qualifications are based on a “dual” apprenticeship, usually lasting 3 years. During this period, apprentices tend to learn both at vocational school and in the company. “Semiskilled workers” refers to a type of non-academically trained workforce that needs no specific occupational competence. Depending on the internal division of labor, sometimes mixed teams of *Facharbeiter* and semiskilled workers can be found.

^5^For details of the methodological approach and its advantages, see Kern and Schumann (1984) and Baethge-Kinsky et al. (2018).

^6^Radio-frequency identification is a technology for contactless identification and localization of objects with the aid of electromagnetic waves.

^7^Discontinuous or batch production here means that the production process is divided into different production stages. Each of them must be completed separately so that the next stage can be initiated.

^8^Continuous production means a production process in which the input materials pass through a highly complex plant. These materials are continuously changed by the chemical and physical processes taking place in the respective subsystems, so that the desired product is produced at the end of the plant.

^9^In Germany, the “Meister” title is given to those completing training lasting several years. It does not indicate a company role.
